# Microbial basis of Fusarium wilt suppression by *Allium* cultivation

**DOI:** 10.1038/s41598-018-37559-7

**Published:** 2019-02-08

**Authors:** Tomoki Nishioka, Malek Marian, Issei Kobayashi, Yuhko Kobayashi, Kyosuke Yamamoto, Hideyuki Tamaki, Haruhisa Suga, Masafumi Shimizu

**Affiliations:** 1 The United Graduate school of Agricultural Science, Gifu University, Gifu, Japan; 2 Center for Molecular Biology and Genetics, Mie University, Tsu, Mie Japan; 3 Bioproduction Research Institute, National Institute of Advanced and Industrial Science and Technology (AIST), Tsukuba, Japan; 4 Life Science Research Center, Gifu University, Gifu, Japan

## Abstract

Crop rotation and intercropping with *Allium* plants suppresses Fusarium wilt in various crops. However, the mechanisms underlying this phenomenon have not been fully elucidated. This study was designed to assess the role of microorganisms inhabiting *Allium* rhizospheres and antifungal compounds produced by *Allium* roots in Fusarium wilt suppression by *Allium* cultivation. Suppression of cucumber Fusarium wilt and the pathogen multiplication by *Allium* (Welsh onion and/or onion)-cultivated soils were eliminated by heat treatment at 60 °C, whereas those by Welsh onion-root extract were lost at 40 °C. The addition of antibacterial antibiotics eliminated the suppressive effect of Welsh onion-cultivated soil on pathogen multiplication, suggesting the contribution of antagonistic gram-negative bacteria to the soil suppressiveness. The Illumina MiSeq sequencing of 16S rRNA gene amplicons revealed that genus *Flavobacterium* was the predominant group that preferentially accumulated in *Allium* rhizospheres. *Flavobacterium* species recovered from the rhizosphere soils of these *Allium* plants suppressed Fusarium wilt on cucumber seedlings. Furthermore, confocal laser scanning microscopy revealed that *Flavobacterium* isolates inhibited the multiplication of the pathogen in soil. Taken together, we infer that the accumulation of antagonistic *Flavobacterium* species plays a key role in Fusarium wilt suppression by *Allium* cultivation.

## Introduction

*Fusarium oxysporum* has an extremely broad range of hosts and is one of the most devastating soil-borne pathogens, causing symptoms such as damping-off, root rot, and vascular wilt in crop plants^1,2^. It can saprophytically survive on soil and plant debris in the absence of a host^3^ and remain viable for a long time by producing chlamydospores, thereby making Fusarium wilt very difficult to control. Although effective control measures for Fusarium wilt include the use of resistant cultivars or rootstock^4,5^, this resistance is often overcome by new races of pathogens; moreover, the development of new resistant cultivars is time-consuming^6^. Another strategy for control includes the fumigation of soil using chemicals, such as chloropicrin^7^; however, this approach often negatively affects the environment as well as human health^8^.

The consecutive monocultures of agricultural crops lead to the accumulation of soil-borne fungal pathogens, including *F*. *oxysporum*^9^. Therefore, crop rotation and intercropping have received increasing attention in recent years because they have potential for managing soil-borne diseases^10–13^. In Japan and China, crop rotation and intercropping with *Allium* plants, such as Welsh onion (*Allium fistulosum*), onion (*A*. *cepa*), and Chinese chive (*A*. *tuberosum*), reportedly prevent the Fusarium wilt of bottle gourds (*Lagenaria siceraria*), spinach (*Spinacia oleracea*), tomato (*Solanum lycopersicum*), and banana (*Musa* spp.)^10,11,14,15^.

Two hypotheses that explain the mechanisms responsible for the suppression of Fusarium wilt by *Allium* cultivation have been proposed. The first hypothesis implicates the involvement of antimicrobial compounds released from roots of *Allium* plants^16^. However, there is limited evidence that the antimicrobial compounds released from the roots of *Allium* plants indeed reduce the incidence of Fusarium wilt. The second hypothesis, based on the known importance of the soil microbiome in the suppression of soil-borne diseases^17,18^, implicates microorganisms associated with *Allium* plants in the suppression of Fusarium wilt. However, although intercropping with *Allium* plants, such as onion and garlic (*A*. *sativum*), changes the bacterial diversity and structure of the soil^19,20^, no explicit evidence indicates that these changes play a role in the suppression of Fusarium wilt. Rhizosphere microbial communities are directly influenced by the root exudates of host plants and differ across plant species^21–23^. Therefore, we hypothesized that rhizospheres of *Allium* plants harbor unique microbial communities and that some of the predominant microorganisms are involved in the suppression of Fusarium wilt induced by *Allium* cultivation.

In this study, we first investigated whether microorganisms inhabiting *Allium* rhizospheres and antifungal compounds produced by *Allium* roots contribute to the suppression of cucumber Fusarium wilt caused by *F*. *oxysporum* f. sp. *cucumerinum* (Focu) isolate GUS77, which was used as a representative pathogenic isolate of *F*. *oxysporum*. We further identified the predominant rhizobacterial groups of *Allium* plants by the Illumina MiSeq sequencing of 16S rRNA gene amplicons. The identified bacteria were then isolated and assessed for their ability to suppress cucumber Fusarium wilt by culture-dependent measures to elucidate the importance of the predominant rhizobacteria of *Allium* plants in Fusarium wilt suppression.

## Results

### Fusarium wilt suppressiveness of plant-cultivated soils and of soil amended with Welsh onion root-extract

**Figure 1:**
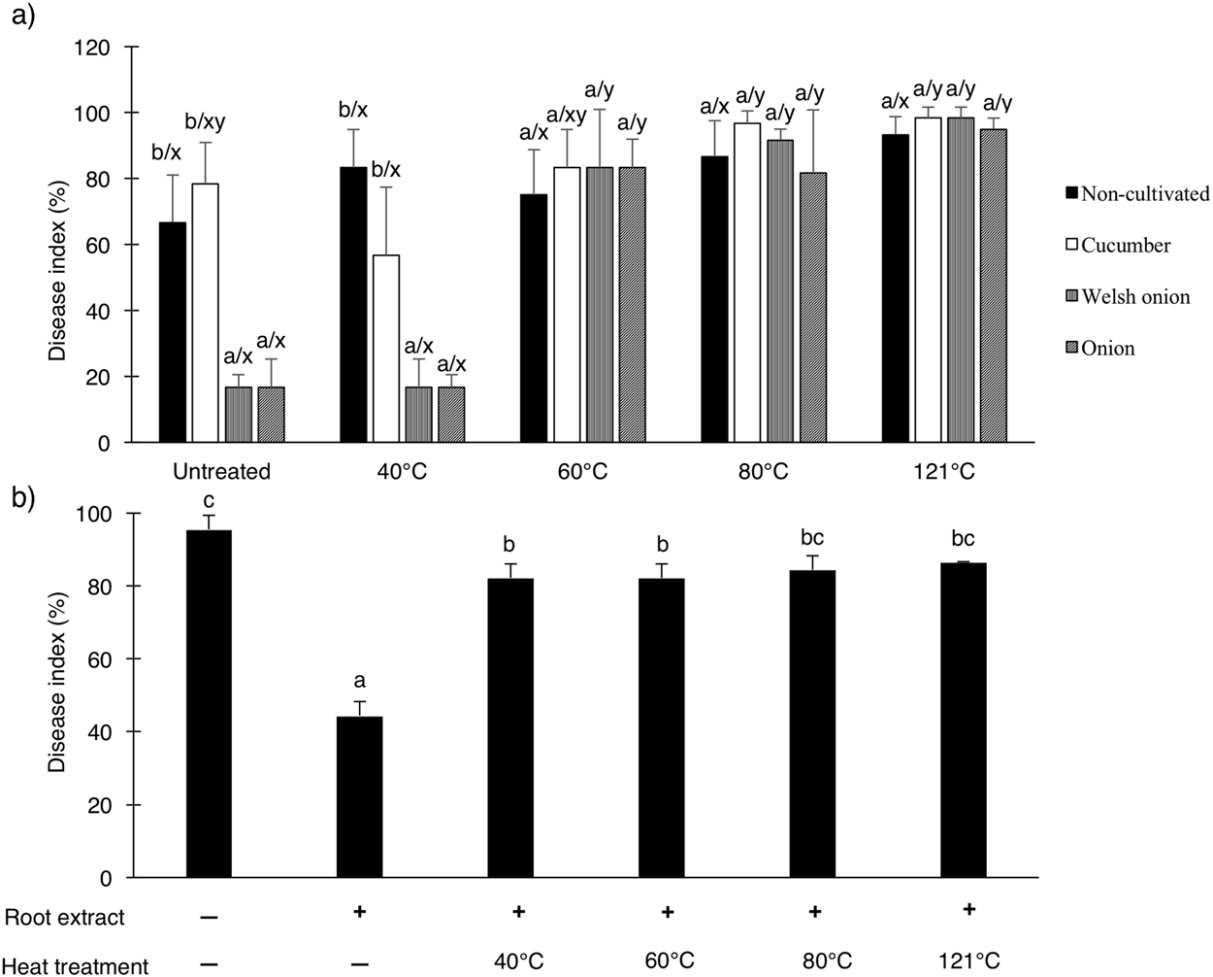
Effect of heat treatment on the suppressive ability of *Allium*-cultivated soils and soil supplemented with root extract of Welsh onion against cucumber Fusarium wilt. (**a**) Disease index in each cultivated soil and non-cultivated soil. Bars represent mean ± SD (n = 4), and the different letters above the bars indicate statistically significant differences between plant treatments (a–b) and between heat treatments (x–y) (*P* < 0.01, Tukey’s test). (**b**) Disease index in soil supplemented with root extract of Welsh onion. Bars represent mean ± SD (n = 3), and the different letters above the bars indicate statistically significant differences (*P* < 0.01, Tukey’s test).

The severity of cucumber Fusarium wilt was significantly reduced in *Allium* (Welsh onion and onion)-cultivated soils compared with non-cultivated soil and cucumber-cultivated soil (*P* < 0.01) (Fig. 1a), indicating that *Allium* cultivation conferred cucumber Fusarium wilt suppressiveness to soil. Similarly, cucumber Fusarium wilt severity in soil amended with an aqueous extract of Welsh onion roots was also significantly lower than that in unamended soil (*P* < 0.01) (Fig. 1b). The suppressive effect of *Allium*-cultivated soils on cucumber Fusarium wilt was not diminished after heat treatment at 40 °C. However, when *Allium*-cultivated soils were treated at 60 °C, 80 °C, or 121 °C, they lost their ability to suppress cucumber Fusarium wilt (Fig. 1a). In contrast, the suppressiveness of soil supplemented with Welsh onion root extract was reduced almost completely after heat treatment at 40 °C (Fig. 1b).

### Impact of heat treatment on culturable bacterial and fungal populations in *Allium*-cultivated soils

The population densities of culturable gram-negative bacteria in non-heat-treated Welsh onion-cultivated soil and non-heat-treated onion-cultivated soil were 0.7–1.3 × 10^8^ and 3.4–6.0 × 10^7^ cfu/g dry soil, respectively (Table 1). Heat treatment at 40 °C did not influence the population densities of culturable gram-negative bacteria. However, the population densities of culturable gram-negative bacteria in *Allium*-cultivated soils treated at a temperature of 60 °C or higher were reduced more than 10-fold compared to the non-heat-treated cultivated soils. Similarly, the population densities of culturable fungi in both cultivated soils decreased sharply when the soil was treated at a temperature ≥ 60 °C. In contrast, the population densities of culturable gram-positive bacteria in both cultivated soils did not change after heat treatment at a temperature between 40 °C and 80 °C.

**Table 1:** Effect of heat treatment on population densities of culturable bacteria and fungi in *Allium* plant-cultivated soils (cfu gram^−1^ dry soil).

Rhizosphere soil	Microorganism	Heat treatment				
		No treatment	40 °C	60 °C	80 °C	121 °C
Welsh onion	Bacteria					
	Gram-negative	0.7–1.3 × 10^8^	0.6–1.1 × 10^8^	<2.8 × 10^6^	ND	ND
	Gram-positive	0.7–1.0 × 10^8^	5.0–9.7 × 10^7^	2.0–3.9 × 10^7^	1.9–3.5 × 10^7^	ND
	Fungi	0.8–3.1 × 10^6^	0.5–2.4 × 10^6^	0.3–1.8 × 10^4^	<1.5 × 10^2^	ND
Onion	Bacteria					
	Gram-negative	3.4–6.0 × 10^7^	2.6–4.7 × 10^7^	<1.9 × 10^6^	ND	ND
	Gram-positive	4.0–6.7 × 10^7^	2.1–3.0 × 10^7^	2.0–3.0 × 10^7^	2.3–2.7 × 10^7^	ND
	Fungi	2.4–2.7 × 10^5^	1.5–1.8 × 10^5^	2.3–3.7 × 10^3^	<2.3 × 10^2^	ND

The culturable bacterial densities were estimated using 1/10 strength tryptic soy agar, and Gram reaction was determined using the KOH method. The culturable fungal densities were estimated using rose bengal-streptomycin agar. The experiment was repeated three times. ND: not detected.

### Inhibitory effect of plant-cultivated soils and root extracts on multiplication of *Fusarium oxysporum*

In Experiment 1, multiplication of FocuGFP-10, a green fluorescent protein (GFP)-tagged isolate of GUS77, in liquid medium was significantly inhibited by the supplementation of soil suspensions, regardless of soil type, when compared with the control (*P* < 0.01) (Table 2). When comparing Welsh onion-cultivated soil with the other two soils (i.e., non-cultivated soil and cucumber-cultivated soil), the inhibitory effect of the former was significantly higher than that of the latter two types of soil (*P* < 0.01). This inhibitory effect of Welsh onion-cultivated soil was not affected by heat treatment at 40 °C. However, when the suspension of Welsh onion-cultivated soil was treated at 60 °C, the inhibitory effect on FocuGFP-10 multiplication was diminished. Additionally, in Experiment 2, treatment with antibacterial antibiotics completely abolished the inhibitory effect of Welsh onion-cultivated soil. In Experiment 3, amendment with Welsh onion root extract also significantly inhibited FocuGFP-10 multiplication in liquid medium compared with the control (*P* < 0.01). In contrast, amendment with cucumber root extract did not affect FocuGFP-10 multiplication. The inhibitory effect of Welsh onion-root extract was abolished after heat treatment at both 40 °C and 60 °C.

**Table 2:** Inhibitory effect of Welsh onion-cultivated soil and root extracts on multiplication of *Fusarium oxysporum* f. sp. *cucumerinum* in liquid medium.

Experiment	Type of amendment in the liquid medium^a^	Heat/Antibiotics treatment	Focu density (log spores ml^−1^)^e^
Experiment 1	Welsh onion-cultivated soil^b^	No treatment	5.76 ± 0.10 a
		40 °C	5.82 ± 0.09 a
		60 °C	6.13 ± 0.02 b
	Cucumber-cultivated soil^b^	No treatment	6.11 ± 0.04 b
	Non-cultivated soil^b^	No treatment	6.21 ± 0.05 b
	Control (SDW)	No treatment	6.58 ± 0.03 c
Experiment 2	Welsh onion-cultivated soil^b^	No treatment	5.78 ± 0.23 a
		Antibiotics^d^	6.30 ± 0.08 b
	Control (SDW)	No treatment	6.77 ± 0.09 b
		Antibiotics	6.63 ± 0.12 b
Experiment 3	Welsh onion-root extract^c^	No treatment	6.01 ± 0.37 a
		40 °C	6.78 ± 0.16 b
		60 °C	6.75 ± 0.16 b
	Cucumber-root extract^c^	No treatment	6.86 ± 0.09 b
	Control (SDW)	No treatment	6.81 ± 0.03 b

^a^Liquid medium: potato sucrose broth including the spores of *Fusarium oxysporum* f. sp. *cucumerinum*.

^b^Welsh onion-cultivated soil, cucumber-cultivated soil, or non-cultivated soil was 1000-fold diluted with sterile distilled water, and then a 0.3 ml of each 1000-fold dilution of soils was added into the liquid medium.

^c^The concentrations of Welsh onion- and cucumber-root extract were 50 mg root material per ml. A 1.5 ml of each root extract was added into the liquid medium.

^d^Antibiotics means a mixture of antibacterial antibiotics comprising ampicillin (300 µg ml^−1^), imipenem (300 µg ml^−1^), and chloramphenicol (300 µg ml^−1^).

^e^Mean ± SD shown (n = 3). For each experiment, figures followed by different letters indicate significant differences (*P* < 0.01, Tukey’s test).

### Microbial community analysis based on 16S rRNA gene amplicons obtained by Illumina MiSeq sequencing

The sequencing resulted in a total of 1,466,567 raw reads detected from 12 soil DNA samples (See Supplementary Table S1). After merging forward and reverse reads using dada2, the number of merged reads for each of the samples ranged from 28,691 to 12,061. The number of OTUs varied among the different samples from 810 to 294. The rarefaction curve suggested that the number of reads was sufficient to assess the diversity of bacteria in the rhizosphere communities (see Supplementary Fig. S1).

**Figure 2:**
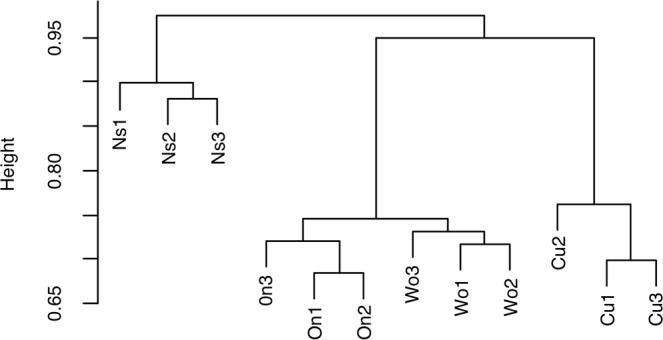
Contrasted clustering of each rhizosphere soil and non-cultivated soil samples based on OTUs composition according to Bray–Curtis similarity. Wo: Welsh onion rhizosphere soil, On: onion rhizosphere soil, Cu: cucumber rhizosphere soil, and Ns: non-cultivated soil. There were three replicates for each type of soils.

The α-diversity (Shannon index) of bacteria of *Allium* rhizosphere (Welsh onion and onion) and cucumber rhizosphere revealed no significant differences, while those of onion and cucumber were significantly lower than that of non-cultivated soil (*P* < 0.05) (see Supplementary Table S1). Clustering analysis based on the OTUs according to the Bray–Curtis index (β-diversity) indicated that bacterial community structures in rhizosphere soils of *Allium* plants were closely related and clearly separated from those in rhizosphere of cucumber and those in non-cultivated soil (Fig. 2). At genus level, the predominant rhizobacterial groups of both *Allium* plants (relative abundance of more than 1.0%) were *Devosia*, *Flavobacterium*, and *Kaistobacter* (Table 3). Among these bacterial genera, only *Flavobacterium* species were significantly abundant in the rhizosphere soils of *Allium* plants compared with cucumber rhizosphere soil and non-cultivated soil (*P* < 0.05).

**Table 3:** Relative abundances of genera comprising more than 1.0% of genera either in each rhizosphere soil (Welsh onion, onion, or cucumber), or non-cultivated soil.

Genus	Welsh onion	Onion	Cucumber	Non-cultivated
*Agrobacterium*	0.3 ± 0.1 b	0.1 ± 0.2 b	1.0 ± 0.3 a	0.0 ± 0.0 b
*Bacillus*	1.7 ± 0.1 a	0.7 ± 0.3 b	2.3 ± 0.4 a	1.9 ± 0.4 a
*Chitinophaga*	0.2 ± 0.0 b	0.2 ± 0.2 b	1.1 ± 0.1 a	0.4 ± 0.3 b
*Devosia*	1.9 ± 0.1 ab	2.4 ± 0.6 a	2.4 ± 0.9 a	0.8 ± 0.2 b
*Flavisolibacter*	0.6 ± 0.3 b	0.5 ± 0.2 b	1.4 ± 0.0 a	0.4 ± 0.1 b
*Flavobacterium*	3.4 ± 0.3 a	1.7 ± 0.4 b	0.2 ± 0.2 c	0.4 ± 0.3 c
*Kaistobacter*	1.3 ± 0.3 a	1.5 ± 0.3 a	2.1 ± 0.9 a	2.1 ± 0.8 a
*Methylibium*	0.8 ± 0.1 b	1.8 ± 0.2 a	0.3 ± 0.3 bc	0.1 ± 0.1 c
*Nitrospira*	0.3 ± 0.1 b	0.1 ± 0.1 b	0.5 ± 0.0 b	1.2 ± 0.3 a
*Novosphingobium*	0.1 ± 0.1 b	0.0 ± 0.0 b	2.0 ± 0.6 a	0.3 ± 0.1 b
*Opitutus*	0.4 ± 0.1 b	0.1 ± 0.1 b	1.6 ± 0.5 a	0.2 ± 0.1 b
*Pseudoxanthomonas*	1.0 ± 0.3 a	0.8 ± 0.1 a	0.9 ± 0.3 a	0.0 ± 0.0 b
*Rhodoplanes*	0.6 ± 0.3 a	0.6 ± 0.1 a	0.6 ± 0.3 a	1.1 ± 0.3 a
*Steroidobacter*	0.5 ± 0.1 b	0.5 ± 0.1 b	1.4 ± 0.3 a	0.6 ± 0.2 b
*Streptomyces*	0.7 ± 0.5 b	1.2 ± 0.2 ab	1.1 ± 0.1 ab	1.8 ± 0.4 a
*Thermomonas*	1.1 ± 0.5 a	0.4 ± 0.2 ab	0.7 ± 0.2 ab	0.2 ± 0.0 b

Mean ± SD shown (n = 3). Different lowercase letters within a row indicate statistically significant differences (*P* < 0.05, Tukey’s test).

### Suppressive effect of *Flavobacterium* isolates on cucumber *Fusarium* wilt

**Figure 3:**
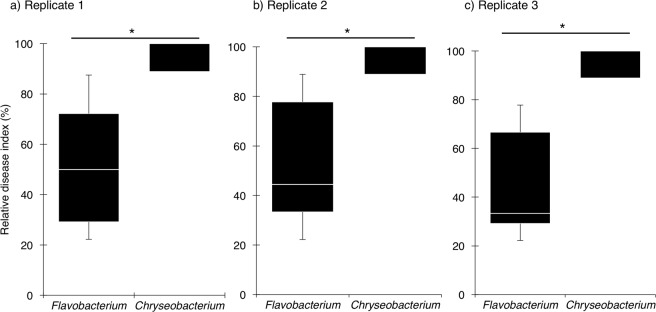
Box-plot showing the suppressive effect of *Flavobacterium* isolates and *Chryseobacterium* isolates against cucumber Fusarium wilt. Nineteen isolates of *Flavobacterium* species and 15 isolates of *Chryseobacterium* species were assessed. The box-plot shows the minimum, maximum, 25–75%, and median values of the relative disease index. Vertical bars extending beyond the boxes represent the 5th and 95th percentiles. Asterisks indicate statistically significant difference (*P* < 0.01, Mann–Whitney U-test).

In replicate 1, the median relative disease index (RDI; %) in the soil treated with *Chryseobacterium* isolates, which served as bacterized controls, was 88.9% (Fig. 3a). *Flavobacterium* isolates obtained from *Allium* rhizospheres exhibited significant suppressive effects on cucumber Fusarium wilt than *Chryseobacterium* isolates (*P* < 0.01). The median RDI of *Flavobacterium* treatments was 50.0%. The similar results were obserbed in each replicate (Fig. 3b,c).

### Inhibitory effect of *Flavobacterium* isolates on hyphal growth of Focu in soil

**Figure 4:**
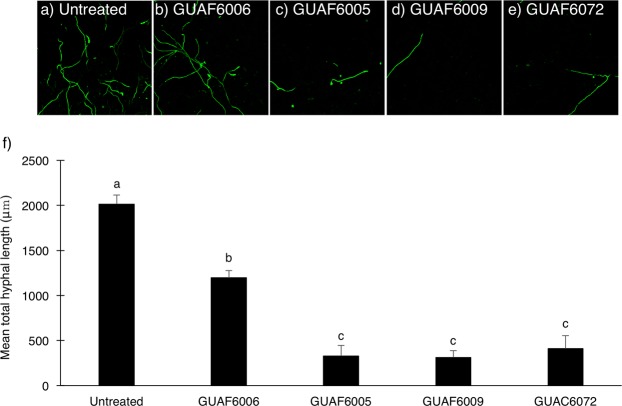
Confocal laser scanning microscopy of FocuGFP-10 in bacterized soil. (**a**) Non-bacterized, (**b**) *Chryseobacterium* isolate GUAF6006, (**c**) *Flavobacterium* isolate GUAF6006, (**d**) *Flavobacterium* isolate GUAF6009, and (**e**) *Flavobacterium* isolate GUAC6072. Data are representative of nine images. (**f**) Mean total length of FocuGFP-10 hyphae in each camera field of view. Bars represent mean of three replications, and the different letters above the bars indicate statistically significant differences (*P* < 0.01, Tukey’s test).

In autoclaved non-bacterized soils, FocuGFP-10 grew hyphae vigorously and the average total hyphal length/camera field reached 2015 μm (Fig. 4). In contrast, hyphal growth was significantly suppressed in soils treated with bacterial isolates, regardless of the bacterial genus (*P* < 0.01) (Fig. 4). However, when comparing *Flavobacterium* and *Chryseobacterium* treatments, the former displayed a significantly stronger inhibitory effect (with the average total hyphal length/camera field ranging from 312 to 411 μm) than the latter (mean = 1195 μm) (*P* < 0.01).

## Discussion

Our study demonstrated that the cultivation of Welsh onion and onion conferred suppressiveness to soil with respect to Fusarium wilt in cucumber plants (Fig. 1a). This result indicates that *Allium* cultivation alters the microbial or chemical properties of soil in a manner that suppresses pathogenic *F*. *oxysporum*. Generally, the role of microorganisms in soil suppressiveness to soil-borne diseases has been made apparent by the fact that the disease suppressive effects of soils are lost upon pasteurization^1,24^. We found that the Fusarium wilt suppressiveness of *Allium*-cultivated soils disappeared after heat treatment at ≥60 °C (Fig. 1a). Similarly, the inhibitory effect of Welsh onion-cultivated soil on the multiplication of Focu in liquid medium was lost after heat treatment at 60 °C but not at 40 °C (Table 2). Heat treatment at 60 °C reduced the population density of culturable gram-negative bacteria and fungi in *Allium*-cultivated soils but not that of culturable gram-positive bacteria, whereas treatment at 40 °C did not affect the populations of those microorganisms (Table 1). In addition, supplementation with antibacterial antibiotics abolished the inhibitory effect of Welsh onion-cultivated soil on Focu multiplication. Taken together, these results suggest that gram-negative antagonistic bacteria accumulated in *Allium*-cultivated soils may be a major factor in cucumber Fusarium wilt suppressiveness.

Cluster analysis based on the data from the Illumina MiSeq sequencing of 16S rRNA gene amplicons demonstrated that the bacterial community structures of *Allium* rhizosphere soils were similar to each other and different from those of cucumber rhizosphere soil and non-cultivated soil (Fig. 2). Interestingly, the gram-negative genus *Flavobacterium* was predominant in (relative abundance >1.0%) and characteristic of the bacterial communities of Welsh onion and onion rhizosphere soil (Table 3). Shen, *et al*.^25^ recently reported that *Flavobacterium* was one of the most abundant bacterial genera present in the soil of banana fields in which Fusarium wilt decline had been occurring. Therefore, we hypothesized that the accumulation of *Flavobacterium* species is a key component of Fusarium wilt suppressiveness of *Allium*-cultivated soils. Indeed, *Flavobacterium* isolates recovered from rhizospheres of Welsh onion and onion exhibited significant suppressive effects against Fusarium wilt on cucumber seedlings (Fig. 3). In addition, isolates having a strong disease-suppressing effect significantly inhibited the growth of Focu hyphae in soil (Fig. 4), suggesting that the accumulation of antagonistic *Flavobacterium* species in soil is an important mechanism of Fusarium wilt suppression by *Allium* cultivation. Although little is known about the suppressive ability of *Flavobacterium* species against soil-borne fungal pathogens, some species of *Flavobacterium* isolated from rhizosphere and soil have been reported to produce antimicrobial substances such as hydrogen cyanide, chitinase, and siderophore^26–28^. We are currently investigating the mode of action of our *Flavobacterium* isolates against Fusarium wilt pathogen.

Shen, *et al*.^18^ reported that bacterial diversity might be an important factor in soil suppressiveness because bacterial diversities were significantly higher in suppressive soils than in conducive soils. However, our data revealed that there were no significant differences in the α-diversity of bacterial communities (Shannon indices) from rhizosphere soils of *Allium* (i.e., Welsh onion and onion) and cucumber (See Supplementary Table S1), indicating that bacterial diversity is not a crucial factor in the suppression of Fusarium wilt by *Allium* cultivation.

Antifungal compounds released from *Allium* roots are recognized to play a role in Fusarium wilt suppression conferred by intercropping or rotation with *Allium* plants^16,29^. In accordance with these reports, the aqueous extract of Welsh onion roots significantly suppressed Focu multiplication in liquid medium and Fusarium wilt on cucumber seedlings (Fig. 1b and Table 2). These suppressive effects of Welsh onion root extract were almost completely lost after heat treatment at 40 °C. These results demonstrated that the suppression of cucumber Fusarium wilt in sterilized soil by supplementation with Welsh onion root extract may be attributed to the direct inhibition of Focu multiplication by heat-sensitive compounds, possibly volatiles, such as 2-methyl-2-pentenal and organosulfur compounds (dimethyl trisulfide, dimethyl disulfide, dipropyl disulfide, and dipropyl trisukfide)^16,30–32^. Zhang, *et al*.^16^ suggested that these antifungal volatiles potentially play a role in the suppression of banana Fusarium wilt when Chinese chive is used as a rotating or intercropping plant. Similarly, Li, *et al*.^12^ found that suppression of Fusarium root rot of peanut by intercropping with a medicinal herb (*Atractylodes lancea*) was mainly due to antifungal volatiles released by the below-ground parts of *A*. *lancea*. However, given that the suppressive effects of Welsh onion- and onion-cultivated soils were retained even after heat treatment at 40 °C (Fig. 1a), antifungal volatiles in root exudate of *Allium* plants may be partially involved but are not a major factor responsible for the suppression of cucumber Fusarium wilt by *Allium* cultivation.

In conclusion, the findings of this study clearly demonstrate that the suppression of Fusarium wilt by *Allium* cultivation is mainly due to the accumulation of antagonistic *Flavobacterium* species. We believe that our results provide important insights into multitrophic interactions among plants, soil-borne pathogens, and natural antagonistic bacteria in crop rotation/intercropping systems. Moreover, we also believe that the elucidation of mechanisms underlying the recruitment and accumulation of antagonistic bacteria by *Allium* plants may lead to the development of novel eco-friendly Fusarium wilt management strategies.

## Materials and Methods

### Preparation of pathogen inoculum

*Fusarium oxysporum* f. sp. *cucumerinum* (Focu) isolate GUS77 used in this study as the challenging pathogen was previously recovered from cucumber fields and was chosen based on its performance on aggressiveness tests. FocuGFP-10, a green fluorescent protein (GFP)-tagged isolate of GUS77, was used for fluorescent microscopy and confocal laser scanning microscopy. Full description is reported in the supplementary information.

### Preparation of soils cultivated with *Allium* and cucumber plants

In order to confirm whether *Allium* cultivation induces soil suppressiveness against pathogenic *F*. *oxysporum*, we evaluated soils cultivated with *Allium* plants or cucumber and a non-cultivated soil. For this, we prepared soils cultivated with Welsh onion, onion, and cucumber (*Cucumis sativus*). Full description is reported in the supplementary information.

### Preparation of root extracts from Welsh onion and cucumber

Root extracts of Welsh onion and cucumber were prepared. Full description is reported in the supplementary information.

### Impact of heat treatment on *Fusarium* wilt suppressiveness by plant-cultivated soils and soil amended with Welsh onion root-extract

Each type of plant-cultivated soil and the non-cultivated soil, prepared as described above, was mixed with double-autoclaved potting soil (Ikubyou-baido: Takii seed and seedling, Kyoto, Japan) and with double-autoclaved river sand in a ratio of 4:1:1 (w/w/w). Six grams of each type of soil mixture were then placed in flat-bottom glass tubes (3 cm in diameter and 12 cm high, Iwaki Glass, Chiba, Japan) and heat-treated in one of the following ways: 1) no treatment, 2) incubation in a water bath at 40 °C for 30 min, 3) incubation in a water bath at 60 °C for 30 min, 4) incubation in a water bath at 80 °C for 30 min, and 5) autoclaving at 121 °C for 60 min.

For the evaluation of soil amended with Welsh onion-root extract, a 1-ml aliquot of the root extract of Welsh onion was added to a flat-bottom glass tube (3 cm in diameter and 12 cm high) containing 6 g (dry weight) of a double-autoclaved soil mixture, prepared by blending field soil sieved through a 2-mm mesh sieve, commercial potting soil (Ikubyou-baido), and river sand in a ratio of 1: 1: 1 (w/w/w). The soil mixture amended with the root extract was then subjected to five types of heat treatments as described above. All soils were then inoculated with Focu by pouring a 4 ml aliquot of a spore suspension (1.5 × 10^4^ spores ml^−1^) into each glass tube and the soil surface was covered with 2 g of sterile vermiculite. Surface-sterilized and pre-germinated cucumber seeds were then planted in this vermiculite layer (1 seed per tube), covered with a small amount of sterile vermiculite, and fertilized with 2 ml of 500-fold-diluted Hyponex solution (Type: 6–10–5, Hyponex Japan, Osaka, Japan). The tubes were then placed in a chamber with controlled conditions (25 °C, 12 h of day light) for 20 days. The severity of the disease developing on the seedlings was assessed using a scale of 0 to 3, where 0 = healthy plant, 1 = cotyledon leaf yellowing and/or vascular browning, 2 = hypocotyl browning, and 3 = seedling dead, and the results were expressed as a disease index (%), calculated using the following formula: {Σ (disease severity of cucumber seedlings)/(the number of replicated tubes × 3)} × 100. There were five replicated tubes (one seedling per tube) per treatment and the experiment was repeated at least three times.

### Impact of heat treatment on culturable bacterial and fungal populations in *Allium*-cultivated soils

In parallel with the assessment of an effect of heat treatment on soil suppressiveness, we investigated possible changes in the bacterial and fungal populations in *Allium*-cultivated soil. Full description is reported in the supplementary information.

### Inhibitory effect of plant-cultivated soils and of the root extract on multiplication of *Fusarium oxysporum*

To clarify whether the accumulation of antagonistic bacteria and/or antifungal compounds produced by *Allium* plants play a role in soil suppressiveness against cucumber Fusarium wilt, the following three experiments (Experiment 1–3) were performed. In Experiment 1, the inhibitory effect of Welsh onion-cultivated soil against Focu multiplication was compared with those of cucumber-cultivated soil and non-cultivated soil. Additionally, the impact of heat treatment on the inhibitory effect of Welsh onion-cultivated soil was also investigated. For heat treatment, 10 g of Welsh onion-cultivated soil, prepared as described before, were suspended in 90 ml of SDW in a 200-ml Erlenmeyer flask and treated at 40 °C and 60 °C, as described above. To evaluate the inhibitory effect of soils against Focu multiplication, a 0.3 ml of each 1000-fold dilution of soils was added into test tubes containing 0.2 ml of potato sucrose broth and 1.5 ml of SDW. Each tube was then inoculated with 1 ml of spore suspension of FocuGFP-10 (3 × 10^5^ spores ml^−1^) and shaken for 16 h at 25 °C on a rotary shaker at 150 rpm. After incubation, the concentration of spores was determined using a haemocytometer under a fluorescence microscope. The spores were counted in five fields of view per sample. There was one tube per treatment and the experiment was repeated three times.

In Experiment 2, the impact of an antibacterial treatment on the inhibitory effect of Welsh onion-cultivated soil was investigated. For the antibacterial treatment, a 100 µl of 100-fold diluted suspension of Welsh onion-cultivated soil was mixed with 900 µl of a mixture of antibacterial antibiotics comprising ampicillin (300 µg ml^−1^), imipenem (300 µg ml^−1^), and chloramphenicol (300 µg ml^−1^), and then incubated for 1 h on a rotatory shaker at 25 °C. The inhibitory effect of the soils against Focu multiplication was tested by the same procedures in Experiment 1. There was one tube per treatment and the experiment was repeated three times.

In Experiment 3, the inhibitory effect of Welsh onion-root extract against Focu multiplication was compared with that of cucumber-root extract. Simultaneously, the impact of heat treatment on the inhibitory effect of Welsh onion-root extract was also investigated. For heat treatment, Welsh onion-root extract was treated at 40 °C and 60 °C as described above. Either 1.5 ml of each root extract (Welsh onion or cucumber) or SDW was added to a test tube containing 1 ml of potato sucrose broth, and this was then inoculated with 500 µl of Focu suspension (6 × 10^6^ spores ml^−1^) and shaken at 150 rpm for 30 h at 25 °C. After the incubation, the spores were counted using a hemocytometer. There was one tube per treatment and the experiment was repeated three times.

### Microbial community analysis based on 16S rRNA gene amplicons obtained by Illumina MiSeq sequencing

This analysis was carried out through the Illumina MiSeq sequencing of 16S rRNA gene amplicons, using soil DNA extracted from non-cultivated soil and from rhizosphere soils of Welsh onion, onion, and cucumber plants. Non-cultivated soil was prepared as described before. Rhizosphere soils were collected from each of three plants of Welsh onion, onion, and cucumber, which were grown in vinyl pots containing field soil for 70 days as described before. The rhizosphere soils and non-cultivated soil were stored at −80 °C until DNA extraction. The DNA extraction was conducted using a FastDNA SPIN Kit (MP Biomedicals, CA, USA) following the manufacturer’s protocol with little modifications for the first step. Briefly, 0.4 g of either each rhizosphere soil or non-cultivated soil was added to lysing matrix tubes containing 878 µl of sodium phosphate buffer, 122 µl of MT buffer, and 100 µl of 20% skim milk solution. The following processes were conducted according to the manufacture’s protocol. The DNA extraction was repeated three times. The V3–V4 region of each DNA sample was amplified with specific primers^33^ and paired-end sequenced following the manufacturer’s protocol (https://support.illumina.com/content/dam/illumina-support/documents/documentation/chemistry_documentation/16 s/16s-metagenomic-library-prep-guide-15044223-b.pdf) on an Illumina MiSeq (Illumina, CA, USA).

Sequence processing was conducted using Qiime2 (version 2018.2) with demux-summarize, dada2^34^, and feature-table. A pre-trained Naive Bayes classifier based on the Greengenes 13_8 99% operational taxonomic units (OTUs) database (http://greengenes.secondgenome.com), which had been trimmed to include V3–V4 regions of 16S rRNA gene, bound by the 465 F/805 R primer pair, was applied to paired-end sequence reads to make taxonomy tables.

Community dissimilarity according to the Bray–Curtis index was calculated based on the OTUs data processed by coverage-based rarefaction^35^ using the pvclust package in R 3.3.1 software. Predominant bacterial groups of rhizosphere soils of *Allium* plants and cucumber, and non-cultivated soil, with relative abundance of more than 1.0% at the genus level were selected. Furthermore, rhizobacterial groups predominant only in *Allium* plants were selected as potential antagonistic bacteria. Rhizobacterial sequence data were deposited in the Sequence Read Archive database under accession numbers DRX121065– DRX121076 (BioProject: PRJDB6419).

### Isolation of *Flavobacterium* species from *Allium* rhizosphere

It was postulated that *Flavobacetrium* species were involved in Fusarium wilt suppression by *Allium*-cultivation. To test this hypothesis, *Flavobacterium* species were isolated from the rhizosphere soils of Welsh onion and onion. Both plants were grown in vinyl pots as described above. Rhizosphere soils of these plants were collected, diluted with SDW, and then spread on the surface of a semi-selective medium (namely PSR2A-C/T agar)^36^ for species of *Flavobacterium* and *Chryseobacterium*. Simultaneously, *Chryseobacterium* species were also recovered from Welsh onion rhizosphere soil. *Flavobacterium* and *Chryseobacterium* species belong to the same family, i.e., *Flavobacteriaceae*.

### Suppressive effect of *Flavobacterium* isolates on cucumber Fusarium wilt

*Flavobacterium* isolates obtained from *Allium* rhizospheres were evaluated for their ability to suppress cucumber Fusarium wilt in the cucumber seedling assay. For comparison, suppressive effect of *Chryseobacterium* isolates obtained from Welsh onion were also tested (designated as “bacterized control”). Full description is reported in the supplementary information.

### Inhibitory effect of *Flavobacterium* isolates on hyphal growth of Focu in soil

To determine whether the *Flavobacterium* isolates could antagonize Focu in soil, hyphal growth in soil bacterized with *Flavobacterium* isolates was examined by confocal laser scanning microscopy (CLSM) using FocuGFP-10 as the challenging pathogen. The soil was bacterized with *Flavobacterium* isolates GUAF6005 (from Welsh onion), GUAF6009 (from Welsh onion), or GUAC6072 (from onion), or with *Chryseobacterium* isolate GUAF6006 (from Welsh onion). The *Flavobacerium* isolates used here exhibited a strong suppressive effect against cucumber Fusarium wilt (RDI < 50%) in the cucumber seedling assay, whereas the GUAF6006 *Chryseobacterium* isolate slightly reduced cucumber Fusarium wilt severity (RDI = 88.9%). As illustrated in Supplementary Fig. S2, a chamber was filled with seven grams of the double-autoclaved soil mixture (field soil: river sand: potting soil = 1:1:1 w/w/w) on a coverglass (Cell Imaging Coverglass 1 Chamber, Eppendorf North America Inc., NY, USA). A small hole was drilled into one of the shorter sides of the chamber. Four milliliters of FocuGFP-10 spore suspension (1.8 × 10^5^ spores ml^−1^) was inoculated into the soil mixture. Subsequently, a 3-ml aliquot of each bacterial isolate (ca. 0.5–1.0 × 10^9^ cells ml^−1^) or SDW was separately applied to the FocuGFP-10-inoculated soil mixtures. Surface-sterilized and germinated cucumber seeds were then planted into the holes (one seed per chamber), and soil surfaces were covered with a small amount of sterile vermiculite. The sides of the coverglasses were covered with aluminum foil and the chambers were incubated in a controlled environmental chamber (25 °C, 12 h of daylight) for 1 week. After incubation, images of FocuGFP-10 hyphae in the soil mixtures were captured at three different locations by CLSM (LSM710, Carl Zeiss, Jena, Germany). The mean of total length of FocuGFP-10 hyphae in each camera field-of-view was then measured using LIA32 software (https://www.agr.nagoya-u.ac.jp/~shinkan/LIA32/). There were three replicates for each treatment.

### Statistical analyses

Statistical analyses were performed using BellCurve for Excel (version 2.13) (Social Survey Research Information, Tokyo, Japan). Full description is reported in the supplementary information.

## Supplementary information

Supplementary Information

## Data Availability

The datasets supporting the conclusions of this study are included within this manuscript and its supplementary files.
