# Supplementary material for: Microbial basis of Fusarium wilt suppression by Allium cultivation
Source: Sci Rep. 2019 Feb 8;9:1715. doi: 10.1038/s41598-018-37559-7 (PMC6368641; doi:10.1038/s41598-018-37559-7)
Supplement: Supplementary file 1 — Supplementary Information [file 41598_2018_37559_MOESM1_ESM.pdf]

Microbial basis of Fusarium wilt suppression by *Allium* cultivation

Tomoki Nishioka[1], Malek Marian[1], Issei Kobayashi[2], Yuhko Kobayashi[2], Kyosuke Yamamoto[3], Hideyuki Tamaki[3], Haruhisa Suga[4], Masafumi Shimizu[1,*]

[1]*The United Graduate school of Agricultural Science, Gifu University, Gifu, Japan*
[2]*Center for Molecular Biology and Genetics, Mie University, Tsu, Mie, Japan*
[3]*Bioproduction Research Institute, National Institute of Advanced and Industrial Science and Technology (AIST), Tsukuba, Japan*
[4]*Life Science Research Center, Gifu University, Gifu, Japan*

* Corresponding author (M. Shimizu)
E-mail address: shimizma@gifu-u.ac.jp
Postal address: The United Graduate school of Agricultural Science, Gifu University, 1-1 Yanagido, Gifu City 5011193, Japan

Supplementary Materials and methods

Preparation of pathogen inoculum

Fusarium oxysporum f. sp. *cucumerinum* (Focu) isolate GUS77 used in this study as the challenging pathogen was previously recovered from cucumber fields and was chosen based on its performance on aggressiveness tests. FocuGFP-10, a green fluorescent protein (GFP)-tagged isolate of GUS77, was used for fluorescent microscopy and confocal laser scanning microscopy. The isolates were pre-cultured in potato sucrose agar (infused potato, 200 g; sucrose, 20 g; agar, 15 g, per 1 liter of distilled water) for 4 days at 25°C before use. Each spore suspension was prepared by inoculating 50 ml of potato sucrose broth (infused potato, 200 g; sucrose, 20 g, per 1 liter of distilled water) with mycelial plugs (8 mm in diameter) obtained from the colony on potato sucrose agar. The inoculated flask was then shaken at 120 rpm for 4 days at 25°C. Thereafter, the culture broth was filtered through three layers of sterile gauze to separate filamentous mycelia from the spores, and the latter was collected by centrifugation of the filtrate at 3,000 rpm for 10 min. These precipitated spores were then resuspended in sterile distilled water (SDW), and the concentration of the suspension was adjusted using a haemocytometer.

Preparation of soils cultivated with *Allium* and cucumber plants

In order to confirm whether *Allium* cultivation induces soil suppressiveness against pathogenic *F. oxysporum*, we evaluated soils cultivated with *Allium* plants or cucumber and a non-cultivated soil. For this, we prepared soils cultivated with Welsh onion, onion, and cucumber (*Cucumis sativus*) as follows. Ninety-day-old seedlings of Welsh onion (cv. Kujohoso), 40-day-old seedlings of onion (cv. Syarumu), and 20-day-old seedlings of cucumber (cv. Tokiwa jibai) were used for transplanting. Each *Allium* plant (three seedlings per pot) or cucumber (one seedling per pot) was transplanted into a vinyl pot (15-cm in diameter, 8-cm deep) filled with soil collected from a field on the experimental farm of Gifu University. The plants were then grown for 70 days in a greenhouse at 20–25°C under natural light with regular watering. After 70 days, the plants were uprooted from the pots and the remaining soil was collected and then sieved through a 2-mm mesh sieve (designated as "cultivated soil"). Additionally, soil maintained under the same conditions without plant cultivation was also prepared (designated as "non-cultivated soil"). Theses soils were used in the following experiments.

Preparation of root extracts from Welsh onion and cucumber

For preparation of root extracts, Welsh onion and cucumber seedlings were grown in vinyl pots containing field soil for 70 days as described above. These plants were uprooted from the pots and the adhering soil was carefully removed from the roots, and then washed with running tap water. The roots were cut off and drained on paper towels. The fresh root weight of each plant and wet weight of the soil remaining in each pot were recorded to determine the fresh root weight (mg) per wet weight (g) of soil. The mean of three replicates was found to be 8.3 and 6.3 mg of

roots (Welsh onion and cucumber, respectively) per g of wet soil. The roots were homogenized in liquid nitrogen using a mortar and pestle, and the homogenate was immediately transferred to a 50-ml tube. To extract aqueous compounds, 30 ml of SDW was added to the tube containing the homogenate and mixed thoroughly with a vortex mixer for 1 min. Thereafter, the homogenate suspension was filtered using filter paper (Advantec Toyo No. 1, Toyo Roshi, Tokyo, Japan) and a micro filter (Millex GV, pore size 0.22 μl, Merck Millipore, Carrigtwohill, Ireland) to remove plant debris and microorganisms. The resulting filtrate was diluted with SDW to a concentration of 50 mg root ml^{-1} and used as root extract. The root extracts were stored at −20°C until use.

Impact of heat treatment on culturable bacterial and fungal populations in *Allium*-cultivated soils

In parallel with the assessment of an effect of heat treatment on soil suppressiveness, we investigated possible changes in the bacterial and fungal populations in *Allium*-cultivated soil. For this, 10 g aliquots of either Welsh onion- or onion-cultivated soil, prepared as described above, were suspended in 90 ml of SDW in a 200-ml Erlenmeyer flask and shaken for 15 min on a rotary shaker at 150 rpm. After shaking, each soil suspension was heat-treated as described above. The population densities of bacteria and fungi were enumerated using the dilution plating method with 1/10 strength tryptic soy agar (TSA; Difco Laboratories, MI, USA) and rose bengal-streptomycin agar[37], respectively. Eleven to 20 bacterial colonies on the plates that yielded 11–100 bacterial colonies on 1/10-strength TSA were picked and purified using quadrant streaking. The Gram reactions of the bacterial isolates were determined using the non-staining (KOH) method[38]. The experiment was repeated three times.

Suppressive effect of *Flavobacterium* isolates on cucumber Fusarium wilt

Flavobacterium isolates obtained from *Allium* rhizospheres were evaluated for their ability to suppress cucumber Fusarium wilt in the cucumber seedling assay. For comparison, suppressive effect of *Chryseobacterium* isolates obtained from Welsh onion were also tested (designated as "bacterized control"). Each isolate of *Flavobacterium* (n =19) and *Chryseobacterium* (n =15) was cultured in Reasoner's 2A broth at 25°C with shaking at 200 rpm for 24 h. Protocol of cucumber seedling assay was same as that used for the evaluation of suppressive effect of the root extract of Welsh onion. Instead of the root extract, a 1-ml aliquot of each bacterial culture (ca. 0.5–1.0 × 10^9 cells ml^{-1}) was applied to the Focu-inoculated soil mixture in flat-bottom glass tubes. As a non-bacterized control, the same volume of SDW was added. After planting cucumber seeds, the tubes were incubated in a controlled environmental chamber (25°C, 12 h of daylight) for 14 days. The disease severity in the cucumber seedlings was assessed using the same rating scale described above, and the results were expressed as a relative disease index (RDI; %), calculated using the following formula: RDI = (mean disease scale in the bacterized treatment/mean disease scale in non-bacterized control treatment) × 100. There were three tubes per treatment and the experiment

was repeated three times.

Statistical analyses

Statistical analyses were performed using BellCurve for Excel (version 2.13) (Social Survey Research Information, Tokyo, Japan). The means of disease index were compared using ANOVA followed by Tukey's test at $P < 0.01$. The data of population density of FocuGFP-10/Focu were transformed into base 10 logarithms and then compared using ANOVA followed by Tukey's test at $P < 0.01$. Relative abundances of genera in each rhizosphere soil and non-cultivated soil were compared using ANOVA followed by Tukey's test at $P < 0.05$. The RDI in the bacterized soils were compared using Mann–Whitney U-test at $P < 0.01$. Means total length of FocuGFP-10 hyphae in bacterized soils were compared using ANOVA followed by Tukey's test at $P < 0.01$.

References for supplementary materials and methods

37. Martin, J. P. Use of acid, rose bengal, and streptomycin in the plate method for estimating soil fungi. *Soil Sci.* **69,** 215–232 (1950).

38. Gregersen, T. Rapid method for distinction of gram-negative from gram-positive bacteria. *Eur. J. Appl. Microbiol. Biotechnol.* **5,** 123–127 (1978).

Supplementary Table S1 Sequencing results and alpha-diversity

Soil sample	Number of raw reads	Number of reads processed by DADA2	Number of OTU	Number of genus	Shannon index
Welsh onion	127248 ± 5780	21378 ± 1474	552 ± 42	80 ± 5	5.87 ± 0.06 ab
Onion	124055 ± 16139	17924 ± 2071	475 ± 31	74 ± 8	5.66 ± 0.04 a
Cucumber	107147 ± 25224	15597 ± 4516	391 ± 116	75 ± 17	5.45 ± 0.25 a
Non-cultivated soil	130404 ± 19476	25645 ± 4397	705 ± 125	83 ± 6	6.24 ± 0.20 b

Data are presented as mean ± SD (n = 3). The values followed by different letters within a row were significantly different according to Tukey's test ($P < 0.05$)

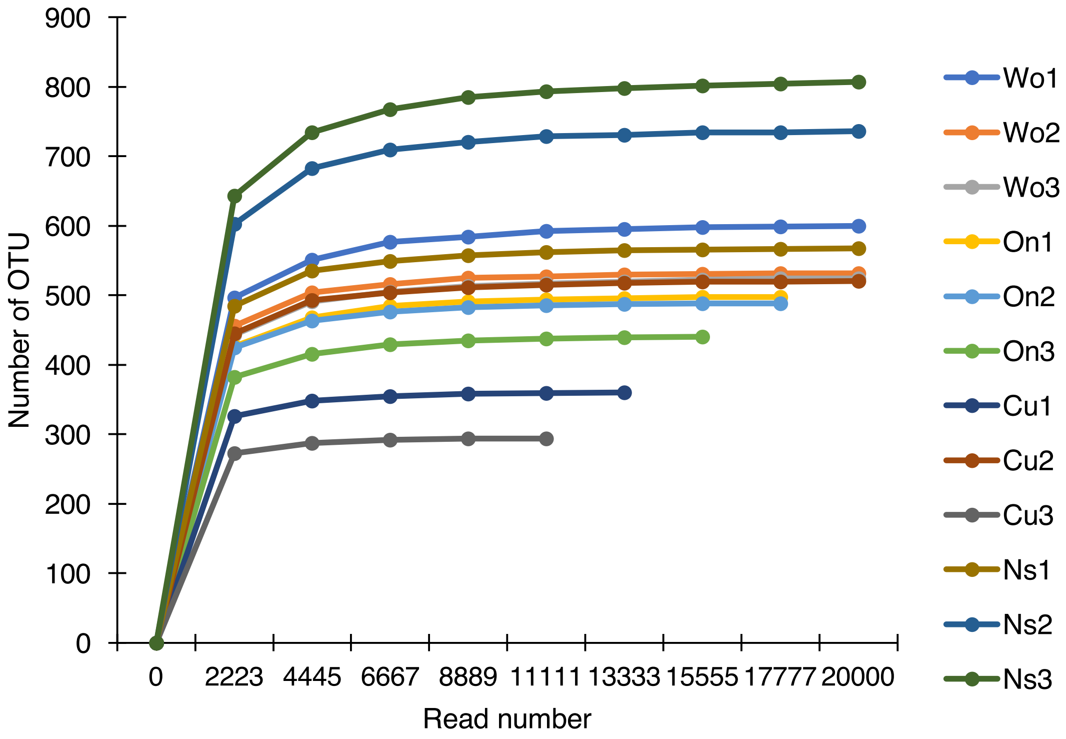

Supplementary Figure S1 Rarefaction curves for microbe operational taxonomic units (OTUs) from each soil sample. Wo: Welsh onion rhizosphere soil, On: onion rhizosphere soil, Cu: cucumber rhizosphere soil, and Ns: non-cultivated soil

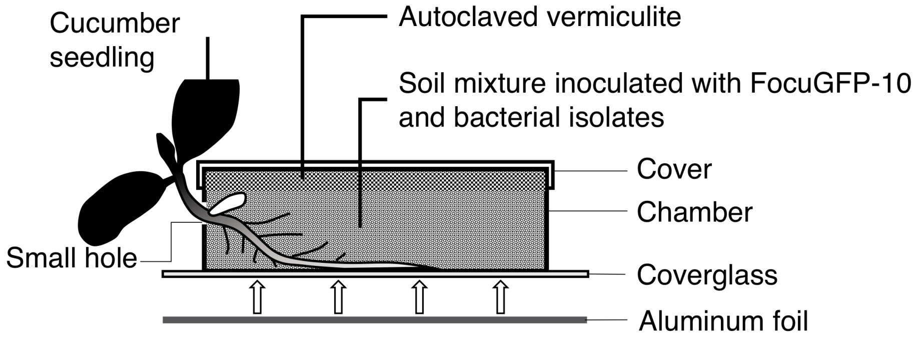

Supplementary Figure S2 Experimental set up used to test inhibitory effect of *Flavobacterium* isolates on hyphal growth of FocuGFP-10 in soil
